# Structure of the Natural Transgene *PgiC2* in the Common Grass *Festuca ovina*

**DOI:** 10.1371/journal.pone.0013529

**Published:** 2010-10-20

**Authors:** Pernilla Vallenback, Lena Ghatnekar, Bengt O. Bengtsson

**Affiliations:** Department of Biology, Genetics, Lund University, Lund, Sweden; Montreal Botanical Garden, Canada

## Abstract

**Background:**

A horizontal gene transfer has brought an active nuclear gene, *PgiC2*, from a polyploid *Poa* species (*P. palustris* or a close relative) into the common grass sheep's fescue (*Festuca ovina*). The donor and the receptor species are strictly reproductively separated, and *PgiC2* occurs in a polymorphic state within *F. ovina*. The active gene copy is normally closely linked to a very similar pseudogene.

**Methodology/Principal Findings:**

By genome walking we have obtained the up- and downstream sequences of *PgiC2* and of corresponding genes in the donor and recipient species. Comparisons of these sequences show that the complete upstream region necessary for the gene's expression is included in the transferred segment. About 1 kb upstream of *PgiC2* a fragment with transposition associated properties has been found (TAF). It is present in *P. palustris* and its polyploid relatives, though not at the homologous position, and is absent from many other grasses, including non-transgenic *F. ovina* plants. It is possible that it is a part of a transposing element involved in getting the gene into a transferring agent and/or into the recipient chromosome.

**Conclusions/Significance:**

The close similarity of the up- and downstream regions with the corresponding regions in *P. palustris* excludes all suggestions that *PgiC2* is not a HGT but the result of a duplication within the *F. ovina* lineage. The small size of the genetic material transferred, the complex nature of the *PgiC2* locus, and the associated fragment with transposition associated properties suggest that the horizontal transfer occurred via a vector and not via illegitimate pollination.

## Introduction

Horizontal gene transfer, HGT, can be defined as transfer of genetic material between distantly related genomes by some mechanism other than sexual fertilization. Originally believed to be very rare or non-existent in higher plants, mitochondrial gene transfers involving several species have now been reported [1]. Recently, it has been claimed that nuclear DNA transfer can also occur [2]–[4]. Some species participating in HGT have a symbiotic relationship [5]–[6], and genes have been recorded to transfer from host to parasite as well as from parasite to host [5]–[6], but such coevolutionary associations do not seem to be obligatory. One of the known nuclear transfers consists of a transposing element [2], but in no case has the exact mechanism underlying a horizontal gene transfer in plants been fully elucidated.

Two major hypotheses exist for how a horizontal gene transfer in higher plants can take place. The first suggests that genetic material is introgressed into a different genetic background after a pollination event between two species. Since by definition the genomes of the two species must be distantly related, such a deviant pollination may not lead to a regular fertilization and may entail disturbances such as chromosome fragmentation [7]. Using wide crosses in combination with embryo rescue and other laboratory technologies, researchers in plant breeding have succeeded in transferring chromosomes, chromosome arms, or parts of chromosomes between species and/or genera [see, for example, 8]. Whether such processes occur in nature is, however, uncertain. If they occur, they would probably involve large chromosome segments and more than single genes, just as in artificial crosses.

The second hypothesis suggests that the genetic material is transferred into the new species by a vector. Viruses, bacteria and insects have been suggested as possible vectors with abilities to transfer small DNA fragments. This hypothesis still requires that the transferred fragment ultimately becomes incorporated into a host chromosome. Transposable elements may be involved in the excision of the gene, as well as in its integration into the host chromosome.

We have previously described a horizontal transfer of a functional nuclear gene, designated *PgiC2*, between two grass species [3]–[4]. The process has moved the gene from a polyploid *Poa* species (*P. palustris* is a likely candidate) into the very common diploid grass sheep's fescue, *Festuca ovina* [3]–[4], [9]. No natural hybrids have ever been found between these widespread and species-rich genera [10]–[11]. The transgene is not fixed in *F. ovina* but is carried by many individuals and reach, for example, a frequency of 6.2% for chromosomes sampled in the south Baltic region [9]. By combined genetic and sequence analysis it has been shown that active *PgiC2* genes found in *F. ovina* are normally closely genetically linked to a very similar pseudogene [12], [3]. However, a single plant has been found with two closely linked active *PgiC2* genes (of allelic forms *b* and *c*) and no pseudogenes, and many plants have no active copies of *PgiC2* but only pseudogenes at the locus [12], [9]. These variants probably arose by unequal crossovers between chromosomes with the standard configuration of one active gene closely linked to one pseudogene.

The F. ovina genome is large, with a 1C DNA content of 4.75 pg [15] or roughly 4.4 billion bp, which makes a strategy of constructing and scanning a BAC-library for the study of the molecular structure of *PgiC2* both time-consuming and costly. We therefore choose to base our characterization of *PgiC2* in *F. ovina* on genome walks out of the gene. The technique is sensitive and error prone, and its application to the present problem is complicated by the fact that the transgenic *F. ovina* plants contain many copies of different *PgiC*-genes. However, by carefully rechecking the results, we have assembled well-ascertained sequences up- and downstream of *PgiC2*, as well as of other *PgiC* genes, that lead to a first detailed description of the structure of this unique transgene.

## Results

**Figure 1:**
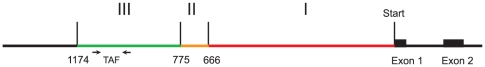
The upstream sequence of *PgiC2*, drawn to scale, with boundaries identified by sequence comparisons with other *PgiC* genes. The Transgene Associated Fragment (TAF) is marked, and the positions of the primers used for its amplification are shown. The results given in Table 1 are coded as follows: Red denotes sequence similarity with all types of *PgiC*-genes. Orange denotes similarity between *PgiC2* and *PgiC* in *P. palustris*. Green denotes similarity between the two tested plants with *PgiC2* with no similarity to any of the other plants.

By genome walking we determined the complete sequence of the *F. ovina PgiC2* gene. In addition, 1337 bp upstream and 604 bp downstream of the gene were identified. Fig. 1 shows the upstream part of the gene, divided into regions with varying degrees of sequence similarity relative to other *PgiC* sequences.

### Comparison between *PgiC2* and Other *PgiC* Sequences

With the same method we obtained 1844 bp upstream sequence of the *F. ovina PgiC1* gene. Table 1 shows the numbers of differences from *PgiC2*. No sequence similarity between *PgiC2* and *PgiC1* could be recognized beyond 666 bp upstream of exon 1. This point defines the boundary between regions I and II in Fig. 1 and Table 1. Within region I the difference between *PgiC1* and *PgiC2* increased with increasing distance from the start codon.

**Table 1:** The difference between *PgiC2* and other sequences over the upstream regions described in Fig. 1.

		Region		
Gene	Exon I	I	II	III
*F. ovina PgiC1*	2/0	47/36	-	-
*P. palustris PgiC*	1/0	12/3	7/4	-
*F. ovina PgiC2 bc*	0/0	0/0	0/0	1/0

“-” implies no significant homology. The first figure gives the number of differences involving single base pair substitutions or indels; the second gives the number of base pair differences or indels that involve more than one consecutive nucleotide.

From *Poa palustris* 2169 bp upstream of the *PgiC* gene were obtained. This sequence was highly similar to the sequence from *PgiC2* (see Table 1), until the similarity abruptly ended at bp 775. This point we take to delimit regions II and III in Fig. 1 and Table 1. No further similarity between the upstream sequences was detected beyond this point.

From the *F. ovina bc* plant, carrying the rare version of *PgiC2* with two active alleles, 1305 bp of the upstream sequence was obtained. Very high similarity was found between this sequence and the sequence from the plant with the standard configuration of *PgiC2* (see Table 1).

### The Transgene Associated Fragment (TAF) and its distribution

Within upstream region III a pair of PCR primers (see Fig. 1) amplified a 145 bp long fragment in the two investigated plants with *PgiC2* genes. A similar band was obtained from the *P. palustris* plant, while the *F. ovina* plant without *PgiC2* did not produce any band. We call this the Transgene Associated Fragment (TAF).

This PCR reaction was also run on 22 *F. ovina* plants from different populations in the south Baltic region, of which eleven contained the *PgiC2* locus and eleven did not. A band of expected length was obtained from all *F. ovina* plants with *PgiC2*, whereas the plants without *PgiC2* did not produce any TAF band. Some of the plants with *PgiC2* had only pseudogenes and no active genes.

To further investigate the distribution of TAF, plants from 14 different grass species were tested with PCR (*P. angustifolia*, *P. nemoralis*, *P. compressa*, *P. chaixii*, *P. supina*, *P. annua*, *F. pratensis*, *F. arundinacea*, *F. polesica*, *Lolium perenne*, *Dactylis glomerata*, *Zea mays*, *Oryza sativa* and *Hordeum vulgare*). Bands of the expected length were obtained from the three polyploid *Poa* species (*angustifolia*, *nemoralis* and *compressa*) but not from any of the other species.

A genome walk in the *PgiC2* plant, starting in TAF and moving away from the gene, resulted in one sequence that was identical to the well-ascertained upstream sequence until base pair 1174. Outside this point a different unique sequence was found. This is the end of region III in Fig. 1 and Table 1.

### Sequence information from downstream *PgiC2*

Downstream of the *PgiC2* gene the sequence was very similar to the corresponding sequence from *PgiC* in *P. palustris*. In 613 base pairs, 15 single base pair substitutions or indels were found plus 12 base pair differences or indels that involved more than one consecutive nucleotide. A comparison with the downstream sequence of *PgiC1* in *F. ovina* gave, however, a completely different result. Here the homology between *PgiC1* and *PgiC2* (and *PgiC* from *P. palustris*) ended immediately downstream the stop codon of the gene.

## Discussion

The *PgiC2* locus in *F. ovina* was originally detected through the presence of too many bands in an isozyme survey [14]. This implies that elements necessary for the expression of *PgiC2*, typically placed upstream of a gene, must have been brought along with it. The sequences of *PgiC2* and *PgiC1* from *F. ovina* and *PgiC* from *P. palustris* are all similar in region I, though *PgiC1* becomes increasingly divergent with increasing distance from the gene. This similarity ends at the boundary to region II, when *PgiC1* becomes different from the other sequences, thereby presumably marking the end of the upstream region necessary for regular gene expression.

The similarity between *PgiC2* and *PgiC* in *P. palustris* extends beyond this point, and includes region II in Fig. 1. This implies that the transfer of genetic material from *P. palustris* into *F. ovina* involved a chromosome fragment that most likely contained not only a structural gene but also its necessary controlling sequences. If *PgiC1* and *PgiC* from *P. palustris* were similar beyond the region of homology with *PgiC2*, then the apparently normal regulation of *PgiC2* expression would need its own separate explanation.

The differences and similarities of these three sequences in the up- and downstream regions give renewed support to and – we would claim – finally prove our earlier conclusion that *PgiC2* in *F.ovina* is the result of a horizontal transfer from *Poa* [3], [4]. The close similarity in upstream regions between *PgiC2* in *F. ovina* and *PgiC* in the distantly related species *P. palustris*, can only be explained by a very recent common history. No evolutionary scenario, involving any kind of stabilizing or directional selection, could lead to the pattern shown in Table 1 and Figure 1 if *PgiC2* were a duplication of the *PgiC1* gene within the *F. ovina* lineage. This conclusion is strongly confirmed by the downstream data. The sequence downstream the *PgiC2* gene is highly similar to the corresponding sequence from *P. palustris* for hundreds of base pairs, while no sequence similarity exists relative to *PgiC1*. Given the complete reproductive separation between the *Festuca* and *Poa* genera [10], [11], and the extensive sequence divergence found between these genera with respect to their standard *PgiC* genes [4] as well as their ITS sequences [12], [13], *PgiC2* undoubtedly represents a case of a horizontal gene transfer, and – as yet – the only example involving a functional nuclear gene among angiosperms [16], [17].

With respect to the question of how this horizontal gene transfer came about, we know as yet too little, but the results from our analysis of the transgene characteristic fragment, TAF, in upstream region III are suggestive. When the end of similarity between the upstream regions of *PgiC2* and *PgiC* from *P. palustris* was found, it seemed reasonable that we had detected the end of the horizontally transferred region and that the “standard” DNA of *F. ovina* “returned” here. To check this suggestion we constructed primers that would amplify only this fragment. However, when targeting these primers to non-transgenic *F. ovina* plants no amplification was detected. Such amplification was only obtained in plants already known to contain the transgenic *PgiC2* gene. From this we conclude that region III in Fig.1 does not correspond to any region in the standard *F.ovina* genome, but must have been brought into this species by the HGT event. In correspondence with this conclusion, the sequence can be found in polyploid *Poa* species.

The TAF sequence has properties associated with transposing elements. As reported above, from the *PgiC2* plant we also obtained a sequence with one end as in TAF and region III but with a completely difference sequence at its upstream end. This result can be taken to imply that TAF exists in more than one copy within the horizontally transferred fragment. It is also notable that TAF resides in *P. palustris* in a different position(s) than immediately upstream *PgiC*, since no trace of it could be detected in the 1844 bp upstream region. Among different grasses, TAF was found in the closely related group of polyploids from which we have earlier shown that *PgiC2* must be derived [4], but not in any of the other tested species. Taken together these data indicate that TAF represents DNA of a mobile nature and that it may have played a role in the HGT process. Further cloning studies to determine the genomic contexts in which TAF are found in different grasses will, thus, be important, as will investigations of its sequence variation (that may cause PCR based methods to fail to detect its presence).

Of the two suggested modes by which horizontal gene transfers may occur – non-standard pollination and vector mediation – we cannot formally exclude the first possibility. We failed, however, to detect any chromosome fragment from *P. palustris* in *F. ovina* plants with *PgiC2* in preliminary experiments with GISH [18], which – if found – would have supported the first alternative. Instead, the small upstream size of the transferred segment (as judged from the comparison with *P. palustris*), the duplication of the *PgiC2* gene at or just after its insertion into a *F. ovina* chromosome (as judged from the similarity between the active and the pseudogene versions of *PgiC2*), plus the fact that it is closely associated with a sequence with transposing characteristics, make us prefer the alternative suggestion that *PgiC2* has been transferred to *F. ovina* by a vector of so-far unknown kind.

## Materials and Methods

### Plant Material, Genome Walking and Sequence Analysis

Sequences of *PgiC2* were obtained from two plants belonging to *Festuca ovina* L. The first had genotype *PgiC1 d/d PgiC2 cφc/*0, the second *PgiC1 d/d PgiC2 bc/*0. The sequence of *PgiC1* was obtained from a *F. ovina* plant with genotype *PgiC1 d/d PgiC2* 0/0. These three plants have earlier been used for descriptions of the *PgiC1*and *PgiC2* genes and for sequence comparisons [4]. The sequence of *PgiC* in *Poa palustris* was based on the same plant as used for an earlier sequence comparison [4].

DNA was extracted using Plant-Mini kit from Qiagen. Genome walking was performed using the Genome walking kit from Sigma-Aldrich according to the suggested protocol in the kit manual. DNA was digested using standard digestion protocols for restriction enzymes. PCR products were ligated into pGEM-T Easy vectors (Promega) and transformed into JM109 Competent cells from Promega. Ampicillin was used for selection. The colonies were used in a PCR reaction with universal primers SP6 and T7. The PCR products were purified and sequenced by Macrogen.

Whenever a new presumptive sequence was obtained, primers were constructed based on the new sequence information and used with a primer inside the well-corroborated sequence in order to validate the result. This was particularly important when the new region was suspected to contain transposable elements. Sequences were aligned and analysed using Sequencher version 4.7. Position numbers were determined from consensus sequences. The up- and downstream sequences of *PgiC2*, *PgiC1* and of *PgiC* from *P. palustris* have been deposited in GenBank (accession no. xx-xx).

The *PgiC2* locus is obviously complex. The plant used as our reference has at least one active gene and one very closely linked pseudogene between which strong sequence similarity holds. With respect to the upstream sequences reported on here, we take them to represent the sequences upstream of both of these genes, since no difference was found between PCR runs with primers specific for the active and the inactive genes. The crucial difference between these genes is instead found in a deletion affecting the boundary between intron 12 and exon 13 [3].

#### Analysis of the Transgene Associated Fragment (TAF)

To test for the presence of the TAF region, DNA from *P. angustifolia*, *P. nemoralis*, *P. compressa*, *P. chaixii*, *P. supina*, *P. annua*, *L. perennes*, *F. pratensis*, *F. arundinacea*, *F. polesica*, *Dactylis glomerata*, *Zea mays*, *Oryza sativa* and *Hordeum vulgare* were obtained from the same plants as used in our earlier sequence comparisons [3]–[4]. For the same purpose additional *F. ovina* plants were taken from the earlier analysed population samples [4] (1 to 4 individuals with and without *PgiC2* from Bornholm, Haget, Eketorp, Mosty and Dresden). The PCR screening was performed using standard method with the TAF primers (xxx) and (xxx).
